# Caring masculinities and the politics of feminist representation in the Portuguese media

**DOI:** 10.3389/fsoc.2025.1557956

**Published:** 2026-01-22

**Authors:** Sofia José Santos, Júlia Garraio, Bárbara Janiques de Carvalho

**Affiliations:** 1 Faculty of Economics, Centre for Social Studies, University of Coimbra, Coimbra, Portugal; 2 Centre for Social Studies, University of Coimbra, Coimbra, Portugal; 3 Faculty of Humanities, University of Coimbra, Coimbra, Portugal

**Keywords:** caring masculinities, media representations, advertisement, gender-based violence, violence prevention, Portugal

## Abstract

In recent decades, the focus on masculinities as a means to sustain or challenge patriarchy has gained significant attention. Concepts such as “hegemonic masculinity”, which embodies the legitimation of patriarchy, and “toxic masculinities”, which encompasses its socially destructive dimensions, have been central to this debate. More recently, “caring masculinities”, have emerged as a potential entry point—not without challenges or ambivalences—for deconstructing patriarchy and the gender-based violence that it perpetuates. The construction and validation of patriarchy have always been closely tied to representations of traditional ideals of masculinity and femininity. Similarly, dismantling patriarchy and gender-based violence is intrinsically connected to representations that foster and uphold gender equality. Given their capacity to create and validate specific representations, media text play a crucial role in shaping, challenging, and reinforcing gender norms and roles. By producing powerful, mobilizing gendered scripts and narratives, media can serve as a transformative tool for either maintaining or disrupting established gender hierarchies. This article focuses on TV advertisements in Portugal to analyze the portrayal of “caring masculinities” and explore how these representations challenge traditional ideals of masculinity. From a collection of 1,602 videos corresponding to TV advertisements for the ten most promoted brands on Portuguese TV in 2015, 247 ads were selected for their alignment with themes of fatherhood, domestic work, and caregiving. Using quantitative content analysis to examine this final corpus of 1,602 videos, this article explores how caring masculinities are represented and negotiated, and discusses how the presence of caring masculinities and their practices, which tend to showcase alternative models of “being a man”, challenge or coopt notions of hegemonic and toxic masculinities—cornerstones of patriarchy and gender-based violence. While some ads present alternative models of masculinity associated with care, emotional availability, and non-violence, others reframe care in ways that reinforce heterosexual, middle-class, and consumerist norms, thereby limiting their transformative potential. The article concludes that media representations of caring masculinities tend to hold both emancipatory possibilities and significant constraints in the sense that while they can challenge aspects of hegemonic and toxic masculinities by legitimising care-oriented practices, they may also co-opt feminist and equality discourses in ways that ultimately stabilise patriarchal structures.

## 1 Introduction

Since the end of the twentieth century, literature on patriarchy and gender equality has provided an extensive discussion on the topic of masculinities with particular emphasis on the work of Connell (2005), who shed light on the nuances of the violence of patriarchy *per se* and, perhaps most importantly, how this violence unfolds and is perpetuated on par and in relation with other systems of privilege and discrimination, namely those based on class, “race”, ethnicity, religion, heteronormativity or age, among others. Concepts such as “hegemonic masculinities”, “subordinated masculinities”, and “marginalized masculinities” were some of her key conceptual proposals.^1^ Through coining different ways of understanding and perceiving masculinities, Connell skillfully underscored that the hegemonic ideal of manhood does not happen linearly but instead combines elements of privilege, oppression and discrimination that extend beyond gender, impacting men and boys and women and girls across their multiple identities, contexts and subsequent power positions.

More recently, the concept of toxic masculinities—a declination of hegemonic masculinity that includes the aspects of hegemonic masculinity that are particularly violent and socially destructive (Kupers, 2005)—has also been included in the literature. While a broad and intersectional lens has been critical in understanding how patriarchal violence manifests and is sustained in societies, to move beyond diagnoses and reconfigure the possibilities that exist to overcome is also an essential step for a feminist agenda. Indeed, as it is consensually understood, a critical outlook on patriarchal masculinities requires a genealogical perspective, a deconstructive analysis and, finally, a propositional perspective. In patriarchal gender norms, in which the masculine prevails over the feminine, issues of care have traditionally been an element that calibrates the binary and hierarchical gender spectrum: when present, care underscores the feminine side of a subject and, when absent, it reinforces the masculine one.

“Caring masculinities” emerged as a conceptual tool for understanding a shifting reality and a means for shaping that change. According to Elliot, the concept of caring masculinities showcases “masculine identities that reject domination and its associated traits and embrace values of care such as positive emotion, interdependence, and relationality” (2016, p. 240). In this sense, caring masculinities contrast with the patriarchal ideal of protection, which has been embodied by archetypes of masculinity such as the bread-winner, the soldier, and the “strong man”. As the head of the family, men have traditionally invoked their role as protectors to claim the right to use violence, if needed, to guide and educate “their” women and children, a situation which—literature shows—continues to shape households across the globe and provoke alarming levels of intimate partner violence (Namy et al., 2017).

Furthermore, underpinning militarism, this ideal of protective masculinity, which has traditionally entitled men to use violence in the name of defending “their land, women and children”, continues to frame contemporary war discourses and to cause so much violence and suffering worldwide, namely against women and children (Enloe, 2023). On the contrary, care is understood as both a conceptual tool and a practice that offers a prism to negotiate and envisage new forms of masculinity that are potentially more inclined to gender equality as it overcomes the feminine exclusive of caring and debunks traditional masculinities. As Cunha et al. (2018) skillfully summarize, “caring masculinities” can, in turn, “be seen as a transgressive social demand that challenges deep-rooted gender stereotypes” (p. 308).

The construction and legitimization of patriarchy are closely connected to traditional representations and imaginaries of masculinity and femininity. As a critical platform for the representation and activation of imaginaries, the media plays a significant role in shaping and influencing gender norms. Indeed, just like literature has consistently pointed out, media may be as pivotal in (re)producing notions of hegemonic masculinity and gender hierarchies as in successfully and cumulatively challenge common sense and deconstruct hegemonic gender norms in societies (e.g., Katz and Earp, 1999). TV advertisements, in particular, are important texts for reinforcing, contesting, challenging and/or producing societal norms and values (Dyer, 1986). Through a strategic articulation between verbal and non-verbal language and, most importantly, through exploring already established networks of meanings, advertising efficiently embodies the politics of representation—even if it is not its primary drive.

If this “double-edged sword” logic marks media representations, the transformation potential of “caring masculinities” in the media may also be entangled in ambivalence: its increasing presence may not necessarily translate into its social normalization since the depictions of caring masculinities are marked by diversity, contradictions and tensions; and, not infrequently, the idea of caring masculinities is dismissed trapping it in humorous plots or labeling it as naivety.

Stemming from the aforementioned ambivalence of both the media power and of the concept and practice of “caring masculinities”, this article approaches media representations of caring masculinities and its potential to debunk patriarchy by analyzing TV advertisement texts of the ten brands that were most advertised on Portuguese TV in 2015, which corresponded to a corpus of 1,602 videos. Portugal is a relevant case study due to its history as a former colonial power, its current EU member-state (where progress in addressing gender-based violence is alongside persisting traditional gender norms), and its position as a semi-peripheral country in international geopolitics and media. As caring masculinities was burgeoning in middle 2010s, the date chosen−2015—reinforces the study's relevance in empirically assessing the conceptual efforts to define this emerging typology of masculinity, which was gaining traction in a context of social transition.

Two thousand and fifteen is also relevant in Portuguese recent history because of particular events and divergent developments that could generate tensions in negotiating gender norms and notions of masculinity. The Socialist Party ended 4 years of a right-wing government by forming a government with the parliamentary support of two far-left parties, the Left Block and the Communist Party. The three parties were committed to deepening the implementation of gender equality and justice. Portugal, one of the first signatories of the Istanbul Convention, has been implementing significant legislation for gender equality. In the 2000s and 2010s, Portugal implemented significant legislation committed to gender equality which challenged hegemonic masculinity and contributed to a redefinition of the traditional family with implications in the understanding of masculinity: access of homosexual unions and single people to artificial procreation (Law No. 32/2006, July 26); sex education in schools (Ordinance No. 196- A/2010, 09 April, which regulates Law No. 60/2009, 6 August, the first legislative document on schools that mentions gender); same-sex marriage (Law No. 9/2010, 31 May); sex change, regardless of the applicant's physiological characteristics (Law No. 7/2011, March 15), sexual orientation and identity (Resolution of the Council of Ministers No. 5/2011); adoption by same-sex couples (Law No. 2_2016, 29 February); right to self-determination of gender identity and gender expression and protection (Law No. 38/2018, 7 August; the law applies to citizens over 16); obligation of all establishments of the educational system, regardless of their public or private nature, to guarantee the necessary conditions for children and young people to feel respected according to their gender identity, gender expression and their sexual characteristics (Order No. 7247/2019, August 16) (Garraio et al., 2023).

Similarly to what was happening in other parts of the world, this kind of legislation, which contributed to the legal recognition of masculinity beyond heteronormativity, became the target of criticism by the increasing campaigns against “gender ideology”. In the case of Portugal, 2013 is a key date: the Portuguese Episcopal Conference had issued the pastoral letter “About Gender Ideology” (*A propósito da ideologia do género*) (November 14, 2013), which constitutes the matrix for the positions taken by the Portuguese Catholic Church regarding already implemented legislation which redefined traditional family and education conceptions (e.g., same-sex marriage, sex change), mentions the “irreplaceable role of the father”, arguing that “affections are not enough to grow: rules and authority are needed, which is accentuated by the role of the father.” The digital newspaper *Observador*, founded in 2014, became a privileged vehicle for the popularization of arguments against “gender ideology” in Portuguese mainstream political debates during the years of socialist rule. With a defined political right-wing trend, *Observador* immediately became a very influential national news media and, as an aggregator of right-wing intelligentsia, a propagator of the country's neo-liberal, conservative and nationalist agendas (Garraio et al., 2023).

To undertake the analysis of our corpus of ads, this article uses quantitative content analysis that used a codebook constituted of two parts, the first on general characteristics of the advertisement and a second one dedicated to mapping and identifying the notions of masculinity and femininity conveyed in the ad, paying particular attention to representations of “caring masculinities”. The article is organized into three sections: the first presents the theoretical and analytical framework that guides our study; the second explores how the politics of representation underpins gender norms and the role advertisement has in this construction; and, finally, the third section presents our study and discusses its results.

The article concludes that Portuguese TV ads depicting caring masculinities frequently integrate elements of hegemonic or toxic masculinities and/or remain largely embedded within patriarchal structures, which reflects a broader societal transition where rigid gender roles are increasingly challenged, yet a clear and widely accepted definition of new masculinities is still being negotiated.

### 1.1 The challenge of caring masculinities amidst patriarchy

Masculinities and femininities are societal constructs that are defined and conceptualized in a binary and relational manner and rooted in overarching patriarchal structures (Connell, 2005). Despite the existence of many definitions of patriarchy, the concept broadly reflects the institutionalization of male dominance within a hierarchical structure, with significant “correlations between one's position and gender” (Galtung, 1996, p. 40), influencing the practice and perceptions of gender-based violence. At the core of patriarchy—sustaining and legitimizing it—lies what Connell coined as “hegemonic masculinity”—a term that denotes the cultural dynamics through which men claim a position of “power over” in the gender hierarchy. It represents a culturally idealized notion of embodying, expressing, performing and behaving as “a real man”, and is generally associated with characteristics such as logical reasoning, control, ambition, aggressiveness, physical robustness, assertiveness, heterosexuality, sexual endurance, sexual entitlement and leadership, to name but a few. The counterweight to hegemonic masculinities is traditional femininities, which constrain being a woman to the private sphere and connect it to traits like submission, care, dependence, fragility, vulnerability and objectification, particularly in a sexual sense (Schippers, 2007).

As patriarchal gender norms are binary, relational and hierarchical, to debunk patriarchy and the gender-based violence it legitimizes, it is paramount to understand and reconfigure the different possibilities that exist revolving the idea of “being a real man”. As it is consensually understood, a critical look at masculinities to challenge patriarchal gender norms and gender-based violence is pivotal. This implies adopting a genealogical understanding of patriarchal relations, a deconstructive analysis, and a propositional look (e.g., Doucet, 2020).

In an attempt to propose alternatives to patriarchal masculinities, care, which was traditionally perceived as an innate and unquestioned responsibility of women (Fine and Glendinning, 2005, p. 602), started to be envisioned as a productive entry point to negotiate and envisage new forms of masculinity that are potentially more prone to gender equality (Scheibling, 2020) and, thus, productive in preventing gender-based violence.

In patriarchal gender norms, care has traditionally contributed to calibrating the binary and hierarchical gender spectrum: when present, care tends to underscore the feminine and, when absent, propends to reinforce the masculine. Consequently, caring masculinities emerge as both a concept and a practice that offers a framework for exploring more gender-equitable forms of masculinity (Hanlon, 2012; Elliott, 2016; Ruby and Scholz, 2018; Doucet, 2020).

Caring masculinities are defined as “masculine identities that reject domination and its associated traits and embrace values of care such as positive emotion, interdependence, and relationality” (Elliott, 2016, p. 240). This typology prioritizes empathy, protection, and support while rejecting aggression and dominance. It underscores the significance of vulnerability, interdependence, and the central role of care in social structures and interpersonal relationships (Elliott, 2016, p. 256).

Due to its proposal, caring masculinities emerge as a significant divergence concerning hegemonic masculinity (Brandth and Kvande, 2018; Roberts and Prattes, 2024). This divergence exists not only because caring masculinities embody a “commitment to care work and gender equality” but also because they “entail a mindful refusal of hegemonic masculinity and inherent prerogatives (privileges, domination, power), as well as of the plural manifestations of “complicit masculinity” that it assumes (Aboim, 2010; Cunha et al., 2018, p. 304). Conceptually, and in practical terms, caring masculinities encompass more than just the act of caregiving and actually aims to be “increasingly realized in the everyday lives of men (e.g., by taking over caregiving tasks in families, by working in ‘feminine' professions of care or through increased self-care consisting of awareness of health or emotional issues, deeper friendships, less risktaking, etc.)” (Scambor et al., 2014, p. 555).

Although caring masculinities have this large conceptual umbrella that points in a counter-hegemonic direction, it is important to perceive nuances within the proposal. The first nuance concerns the concept itself. Different authors point to the fact that caring masculinities encompasses two interconnected dimensions and expressions: “care as work”, which can also be referred to as “care for” and ultimately as “commodification of care (i.e., the practical tasks of care that can also be seen as work), and “care as feeling” or “caring about” (i.e., the emotional and affective dimensions of care) (e.g., Ungerson, 1997, 2005; Elliott, 2016). The development of caring masculinities can begin as a practical and pragmatic approach that gradually evolves into a more affective dimension. The transformation of masculinities toward the inclusion of ethics and practices of care can begin with “caring for” (i.e., engaging in care work) since “when men care for, they can begin to develop the affective, emotional aspects of care” (Elliott, 2016, p. 248–9; 255). This possibility of progression expands the scope and potential effectiveness of caring masculinities as a means of challenging and deconstructing patriarchal structures. Moreover, as Doucet (2020) argues, the coexistence of both dimensions of care among men and women is not a recent phenomenon even if it did not constituted the main trend; for example, in periods of stricter patriarchy, such as the 1930s, some men participated in care work while some women assumed economic provider roles. This dual conceptualization of care, its non-linear relationship with gender roles, and the possibility of starting producing caring masculinities through care work expand the conditions and subjects through which caregiving masculinities can be constructed and legitimized.

The second nuance concerns the fact that caring masculinity can itself validate patriarchy. Indeed, despite the fact that caring masculinities are potentially productive in debunking patriarchy, the disruptive nature of the concept and practice should not allow its limitations and potential contradictions to be overlooked. Research on how primary caregiving fathers navigate social norms concerning masculinity argues that ideas surrounding “caring masculinity are better understood as a broadening of hegemonic masculinity rather than an entirely new or distinct form” (Hunter et al., 2017, p. 1). In practical terms, although there have been significant strides in “parenting practices, identities and values, largely in response to challenging public policies that have been pushing men into early childcare”, in other spheres of the lives of men, namely “in their practices and identities as pupils, workers and in self-care” to embody “caring masculinities remains an extremely incipient enterprise full of obstacles, lacking social and public visibility and awareness” (Cunha et al., 2018, p. 324). This is mainly the result of a combination of gendered expectations and notions of masculinity: men are “more likely to come and go in the realm of unpaid work” while keeping on accepting their privileged position in the labor market; men's lack of interest in “pink-collar” professions, and a sense of pre-determined masculine destiny that still distances themselves from care (Cunha et al., 2018, p. 324, 325). In terms of implications, men can embody different roles, and caring masculinities are not simply a form of being a man that is linearly opposed to hegemonic masculinities. Caring and hegemonic masculinities can coexist within the same individual (Nayak, 2023).

Finally, from an intersectional perspective, caring masculinities are not necessarily fundamentally positive, as their potential can be undermined in neoliberal societies, where caring responsibilities can be commodified and outsourced, often reinforcing class and racial stereotypes, for example (Nayak, 2023). In addition, research into far-right parties has drawn attention to the complex gender roles mobilized and embodied by far-right politicians like Matteo Salvini and activists, whose public performance of caring masculinities coexists with their promotion and normalization of exclusionary policies, hate speech and violence toward immigrants (Scrinzi, 2023).

These ongoing gender negotiations and the selective adoption of specific traits into men's identity, behavior and aspirations point to what the literature usually labels as “hybrid masculinities” and which (Bridges and Pascoe 2014, p. 246) define as “selective incorporation of performances and identity elements associated with marginalized and subordinated masculinities and femininities”. Nowadays, and based upon previous discussion in this article, caring masculinities and hybrid masculinities tend to walk hand-in-hand. According to Connell and Messerschmidt (2005), despite combining hegemonic and non-hegemonic conceptions and elements of masculinities, hybrid masculinities do not necessarily challenge in successful ways patriarchy. Indeed, the concept of hybridity, while indicative of negotiation and seemingly encompassing a partial deconstruction of patriarchy, can paradoxically serve to reinforce patriarchal norms. Bridges and Pascoe (2014) illustrate their point using an example from Bridges himself in 2010: the male participation in the “Walk a Mile in Her Shoes” marches. These events raise awareness about domestic violence by having men wear high-heeled shoes and walk a mile. However, the patriarchal humor associated with men dressing as women reinforces gender inequality, which in turn contributes to the persistence of domestic violence.

Caring masculinities is, thus, an entry point and an important tool to challenge patriarchal assumptions of masculities and feminities.

### 1.2 Advertisement, masculinities and (gendered) politics of representation

In advertising, the main objectives are branding and selling (Lendrevie and Lévy, 2014), achieved through a carefully crafted staged framework that considers the product, the audience, and the goal established. This objective can range from giving meaning to brands, captivating the audience with an imaginary and desired world, stimulating curiosity, evoking emotions that promote the urge to consume, and ultimately fostering consumer loyalty (Caro Almela, 2009). Advertising increasingly relies on a strategic combination of semiotics and linguistics—i.e., compelling visual, sound and textual rhetoric—to explore, in a shortcut logic, existing networks of meanings and cultural associations and, thus, make, with short messages, showcase the qualities and attributes of the products they promote while striving to make these properties meaningful to the public (Schroeder and Zwick, 2004). Through strategic and consumption-driven communication, advertising constructs ideology by validating and contesting societal norms and codes—and/or introducing new ones—through texts and images (Dyer, 1986). In this sense, advertising embodies politics of representation—even if it may not be its primary purpose or drive.

In the West, hegemonic masculinities have prevailed in advertisement media texts. This means that men tend to be depicted as vigorous, rational and logical, powerful, forceful, and assertive, with high heterosexual performances, and independent breadwinners (Schneider and Schneider, 1979; Katz and Earp, 1999; Femiano and Nickerson, 2002; Stern and Mastro, 2004). This has been done based mainly on explicit “stereotyped iconography of masculinity and femininity” (Schroeder and Zwick, 2004, p. 21, 22) but also on more subtle moves like “gender differentiation of voice-over, dominant product-user, and main character”, for example (Maker and Childs, 2003, p. 72).

As gender norms and roles started to change, and, for example, other forms of masculinity started to be equated and represented, gendered representations in advertisements also began to take place (e.g., Allan and Coltrane, 1996; Scharrer et al., 2006; Grau and Zotos, 2016). Considering the representations of men, this change is more recent than changes in women's (Gentry and Harrison, 2010) and tends to occur not necessarily through a logic of disruption but rather in a continuum/negotiation logic (e.g., Santos and de Carvalho, 2018; Leader, 2019). Also, as advertising targeted at men to challenge gender stereotypes and promote gender equality became more frequent, the term “menvertising” started to emerge in an attempt to synthesize these new practices and products (Pando-Canteli and Rodriguez, 2021; Knutson and Waldner, 2017). However, it is worth noting that while “menvertising” has had positive effects on both brands and female audiences, men are reported to show reluctance to accept these new scripts or to dismiss them actively (Knutson and Waldner, 2017; Leader, 2019; Pando-Canteli and Rodriguez, 2021) as men's “starting point is that of privilege” and the loss of that position and the advantages associated with it tends to be met with resistance or discomfort (Pando-Canteli and Rodriguez, 2021, p. 500).

It is also important to note that, while constituting a challenge to traditional gender norms, moves in ad media texts toward gender equality also kept on showing ingrained sexist stereotypes, shedding light on the non-linearity and complexity of gender representations and gender equity. In these cases, men are portrayed as the objects of laughter, fun and mockery, while women are depicted as powerful, attractive subjects. It is also important to note that although men's bodies are becoming more sexualized—just like women's bodies have for a long time—this is done differently from women's sexualization as the role concerning who is the subject and the object sticks to traditional understandings of men and women (Gill, 2008).

## 2 Caring masculinities across TV advertisements in Portugal

Portugal, as a former colonial metropole and an EU member, offers a promising case study to approach these problematics: (i) as a semi-peripheric country both in international geopolitics and in the media, it is a recipient of cultural goods, entertainment and information provided by audiovisual companies, international news agencies and leading international news broadcasters, thus participating in the (re)production of narratives circulating in global media; (ii) it is a minor actor in leading international organizations engaged in the promotion of gender equality policies; (iii) it is a country which witnessed significant advancement in policies addressing gender equality (for instance, regarding paternity leave) and gender-based violence but where the pervasiveness of traditional gender norms in society contributed to the persistence of notions of hegemonic masculinity; (iv) it is a traditionally conservative country regarding sexual morals but with very progressive legislation regarding LGBT+ citizenship, where anti-gender ideology campaigns have been gaining ground precisely around contestation of LGBT+ citizenship (Garraio et al., 2023). Alarming figures of domestic violence and femicide and a significant pay gap are just some of the most visible nefarious consequences of the persistence of traditional understandings of masculinity [Comissão para a Cidadania e a Igualdade de Género (CIG), 2017, p. 8; Neves et al., 2022].

Portugal follows a broader trend observed in Western societies, where advertising continues to perpetuate gender stereotypes, even if in a more subtle and nuanced way. While the overtly rigid gender roles of the past have softened, advertising still reinforces traditional norms, though with a slightly more complex interplay between the negotiation of gender expectations—albeit in a marginal, often residual form. Studies conducted in the late 2000s and early 2010s show a recurring pattern: women are predominantly depicted in nurturing, emotional roles that tie them to family and domesticity, whereas men are more often portrayed in professional settings, occupying the public sphere of work and authority (Pereira et al., 2013; Pereira and Veríssimo, 2008).

In this ongoing reinforcement of gender stereotypes, Fonseca's study on masculinities and the Anthropocene sheds light on how Portuguese advertising has strongly masculinized “red meat,” particularly in its preparation and consumption. The portrayal of meat in this context serves as a symbol of traditional masculinity, reinforcing hegemonic ideals of male power and strength. Even when these messages are communicated subtly—through imagery and subtext—, their impact on shaping everyday masculine practices remains significant (Fonseca, 2017).

Further supporting this view, Januário's research (2016) on advertisements in Men's Health, GQ Portugal, and Max Men magazines from 2011 highlights a striking trend: young (ages 25–35), Caucasian men are overwhelmingly presented with muscular, idealized body types. These representations endorse an “ideal man” in alignment with conventional, dominant notions of masculinity—where whiteness, physical strength, appearance, and fitness are closely tied to male identity.

However, despite this enduring prevalence of traditional masculine ideals, some studies indicate the presence of emerging forms of masculinity in Portuguese advertisements. (Santos and de Carvalho 2018), in their analysis of the “It's Manly!” campaign launched by L'Oréal in Portugal, note a significant shift in how men are represented in ad media texts. While the campaign attempted to challenge conventional gender norms by presenting a more varied and diverse image of masculinity, it still leaned on traditional, and sometimes toxic or harmful, depictions of male robustness and stoicism. In other words, despite the occasional introduction of more progressive imagery, analyzed advertising in that study continued to perpetuate gender inequality by relying on long-established, culturally ingrained representations of masculinity. This points to a hybridity trend that makes the same advertisement or campaign merge texts and subtexts that break with gender norms with those that fall short of disrupting patriarchal structures.

## 3 Methods and corpus of analysis

This article explores how caring masculinities are represented and negotiated, aiming to understand how the media presence of caring masculinities is challenging (or not) notions of hegemonic masculinity by disseminating non-harmful models of masculinity. The selected corpus of analysis includes the video advertisements of the ten brands that were most advertised on Portuguese TV in 2015: Nivea, Danone, Vodafone, MEO, Continente, Lidl, Worten, Intermarché, Securitas Direct, and Nos. The choice for the criteria of the 10 most advertised brands was based upon existing research that, inspired by Cultivation Theory (Gerbner, 1998), suggests a link between repeated exposure to stereotypical portrayals of gender identities and roles and how audiences perceive them (e.g., Pike and Jennings, 2005; Scharrer et al., 2006, p. 219). CISION, an outsourced media clipping company through public procurement, extracted data. The analysis corpus was then narrowed to those that covered the topic of “Fatherhood, domestic work and care”. This was understood as advertisements whose scripts included male identities associated with fatherhood, domestic chores and caring roles or in which the role of father and/or male caregiver was highlighted. While domestic chores and caregiving are traditionally perceived as women's tasks, fatherhood's meaning in the spectrum of masculinities is far more complex: on the one hand, it is a key characteristic of the patriarch in the sense of being the “head of the family”; on the other, being a father implies that one is defined by the relation to the others and brings; the concept also brings connotations of care. Hence, our selection of ads based on the elements of fatherhood, domestic chores and care implied an examination of elements traditionally associated with women, but also an examination of the possible reconfiguration of components traditionally associated with men. The ads containing the three elements corresponded to 15.41% of the overall corpus and included 247 videos. The retrieved data was analyzed through quantitative content analysis following the Codebook created for the study, which included two parts: the first focused on general characteristics of the advertisement (e.g., product's category, imaginaries and narratives contained in the ad), and the second focused on masculinities, fatherhood and caregiving, exploring whether the analyzed advertisement covered explicitly the topic of masculinities, to which role and characteristics it associates being a man to. This method allowed us to map existing data and provide a snapshot of trends in how caring masculinities, understood as a counter-hegemonic and feminist model of “being a man” is represented and negotiated in TV advertisements and, specifically, on how they depictions may co-habit, challenge or even reinforce traditional notions of hegemonic and toxic masculinities, which are key pillars of patriarchy and gender-based violence.

## 4 Data and discussion

This section presents the collected data and, following the codebook, examines how caring masculinities are represented and negotiated. It explores their potential to challenge or reinforce hegemonic and toxic masculinities within patriarchal structures, supported by examples from specific advertisements.

### 4.1 Brand sectors

As described in Table 1, the vast majority of advertisements analyzed fall into the category of supermarkets, representing 82.98% of the total corpus. On the one hand, supermarkets point us to a domain associated with tasks, roles and responsibilities that have been historically linked to femininities and conservative ideals of women: wives, mothers, and female domestic workers were the ones who traditionally went to the grocery and the market to buy what was needed to care for the family and the household. Against this backdrop, representing men engaged in caring activities in supermarkets challenges fixed gender roles. In this context, the depiction of caring masculinity represents, therefore, a productive means of fostering gender equality. By portraying men in spaces traditionally coded as feminine, advertisements create opportunities for the negotiation of gender norm's, contributing to deconstruct patriarchy can cumulatively foster change in society. However, the emphasis on supermarket settings in advertisements depicting caring masculinities and fatherhood introduces additional layers of meaning: men are depicted not only as caregivers but also as consumers with financial control over the household purchases, reinforcing their role as economic providers and thereby aligning with traditional gender norms. Thus, by depicting men within a framework of financial authority, these advertisements echo notions that are part of the repertoire of hegemonic masculinity, subtly reaffirming traditional gender roles. In sum, the supermarket emerges as a dual space—one that simultaneously challenges patriarchal beliefs and ideals by incorporating men into caregiving roles and validates them through the reinscription of hegemonic masculinity in a new context, where particular aspects of it can still be performed.

**Table 1:** Number of advertisements across the different sectors with scripts aligned with ideas of fatherhood, domestic work and care.

**Brand sector**	**Brand**	**Advertisements with scripts aligned with fatherhood, domestic work and care**	**Frequency (*N* = 247)**
Supermarket	Continente	72	29.14
Supermarket	Intermarché	64	25.91
Supermarket	Lidl	69	27.93
Electronic devices and similar	Worten	18	7.28
Telecommunication	Vodafone	9	3.64
Telecommunication	Altice Portugal (Meo)	5	2.02
Telecommunication	NOS	5	2.02
Food industry	Danone	5	2.02
Cosmetics Industry	Beiersdorf (Nivea)	0	0
Security and Maintenance	Securitas Direct	0	0
Total		247	99.97

The supermarket category is followed by the telecommunications sector, which includes internet and telephone services, and then the electronic devices segment, encompassing items like cell phones, washing machines, televisions, and notebooks. Telecommunication and electronic devices have been traditionally associated with masculinity. Even though products like fridges and washing machines refer to domestic chores which have been historically associated with femininity (the “good housewife”), the pervasive association between masculinity and technology—and the traditional stereotype that women are less gifted in science—puts the purchase of these products (the skill to choose the best machine), even if they are to be used by women, in the domain of masculinity.

Advertisements for the food industry—including meat, fish, yogurt, chocolate, coffee, eggs, cakes, ice cream, and unbranded foods like fruits and vegetables—account for only 5% of the corpus. Food is a domain traditionally associated with women's chores: buying the proper ingredients and knowing how to cook and prepare meals were traditionally skills that every “good wife and good mother” should have. Notably, none of the analyzed videos featured advertisements from the cosmetics industry^2^ a sector deeply associated with femininity. Another absence is the security and maintenance sector,^3^ which tends to be associated with men's social roles.

In a country deeply shaped by conservative morals and rigid gender roles, one might expect a scarcity of advertisements featuring men in domains traditionally associated with femininity, such as food and cosmetics. This same traditional cultural background also explains the strong representation of men in technology-related advertisements, as technology aligns with traditional masculine imaginaries. Conversely, the absence of security and maintenance sector ads in the corpus may be attributed to the lower market value of these industries in advertising. What stands out in the table is the surprisingly high number of ads related to fatherhood and caregiving in the supermarket category, a category historically associated and coded as feminine. This prevalence can be interpreted as simultaneously surprising (men are represented in a traditionally domain of women) and predictable (supermarkets also provide a setting where men can still enact traditional masculine roles, such as financial provision). Supermarkets simultaneously offer the possibility of disseminating new forms of masculinity and performing gender equality while (re)enacting elements from hegemonic masculinity, i.e., they can be seen as spaces par excellence for (re)negotiating masculinity and blurring the lines of gender roles. Therefore, supermarkets present opportunities and challenges in promoting caring masculinities and shedding light on the possibilities of hybridity and negotiation of the politics of representation amidst advertisement texts and subtexts.

### 4.2 Narratives and imaginaries

The discussion of Table 2 has to be informed by the following: Although advertisement videos usually have a short duration, they are not restricted to a single narrative, nor do they activate a single imaginary. Identifying only one imaginary or narrative in an advertisement would overlook the plethora of imaginaries that might coexist in a specific media text. This diversity is pivotal it plays a key role in negotiating social norms, allowing for more nuanced representations and, thus, norms negotiation. This means the same ad may have been codified more than once in Table 2.

**Table 2:** Narratives and imaginaries activated by advertisements with themes aligned with fatherhood, domestic work and care.

**Narratives and activated imaginaries**	**Number of narratives and imaginaries**	**Frequency (*N* = 579)**
Family	202	34.88%
Money saving	175	30.22%
Entertainment, leisure and fun	106	18.30%
		1.38%
Emotions and feelings	54	9.32%
Domestic work	25	4.31%
Health, pleasure and wellbeing	8	1.38%
Cars, DIY projects and garden	6	1.03%
Social project or social cause	3	0.51%
Professional realm	0	0%
Total	579	99.95%

As described in Table 2, the vast majority of imaginaries that were activated in the corpus alluded to or recovered imaginaries related to “family”, accounting for 34.88% of the advertisements (advertisements that included family moments, parents, sons and daughters, grandparents, grandsons and granddaughters, siblings, caring for children, children and young people playing, concerns about family members, etc.). This is closely followed by “savings” at 30.22% (understood as advertisements focused on the prices of products or services, reduced prices, promotions, sales, percentage of discounts, etc.). The family and money saving enable the deployment of elements from patriarchal imaginaries—the family provider and protector, the head of the family, the patriarch -which is activated though in conjugation with the imaginary of care—the man who invests in his connection with others and their wellbeing. Like supermarkets, these are narratives prone to hybridity and the renegotiation of hegemonic masculinity through the inclusion and reframing of care.

The third category—entertainment, leisure and fun at 18.30% (i.e., people having fun, friends, games, cinema, beach, dancing, party, moments of relaxation and joy, etc.)—also opens up for hybridity, albeit in a pretty different way. While family and savings are associated with traditional values and responsibility toward others who depend on us (children and elderly people), the third invokes hedonism, having oneself as the priority, and the freedom to indulge oneself without being accountable to others. These are, in fact, two central spheres of twenty-first century neo-liberal consumer societies: on the one hand, the production of a social morality through the family and, on the other, individualism and the pursuit of money for personal satisfaction.

The less prominent categories—emotions and feelings (9.32%), domestic work (4.31%), and hobbies such as cars, DIY projects, and gardening (1.03%)—invoke elements from the repertoire of hegemonic masculinity. The first two reinforce traditional gender binaries, positioning men as rational in contrast to emotional women and framing the household as a feminine domain. The third, on the other hand, aligns masculinity with technology and manual skills. All three, however, contribute to the reinforcement of the abovementioned enactment of neo-liberal consumer values: the narratives of emotions and domestic work put men in relation to others and favor the discursive creation of hybrid masculinities, while the hobbies reinforce the narrative of individualism and hedonism. In contrast, narratives of political and social engagement for the community—often associated with calls for social transformation and questioning the status quo—are underrepresented in the table (0.51%). Men are situated in the private realm as family men taking care of others (especially those more vulnerable like children and elder relatives) or as hedonists taking care of themselves (in the sense of their pleasures).

Notably absent from the discourse are representations related to the professional domain, highlighting a gap in the conversation about fatherhood and caring masculinities in the workplace or the common dissociation between caregiving and professional realms.

The analysis of the gender dynamics underpinning (Table 2) reveals significant insights into the complex negotiations surrounding gender representation. It is essential to highlight that, even though there are efforts to include diverse representations of masculinities, these alternative views are often still connected to traditional ideas about being a man. This connection can manifest in two main ways: either they are confined to the domestic space where the man can still be represented and perceived as the leader, or they remain closely tied to conventional roles, such as that of the economic provider who looks for money saving as a way of making domestic financial management more efficient. Furthermore, renegotiating new elements that contribute to the emergence of hybrid masculinities operates through particular frames of twenty-first century neoliberal societies: the atomization of the social through its reduction to the family and the pursuit of personal pleasure.

### 4.3 The characteristics of “being a man”

The final research question that our analytical categories sought to explore was the roles and characteristics that selected media texts attributed to “being a man” (Table 3). Consistent with the portrayal of imagery and narratives, it is common to observe multiple representations of masculinity within a single advertisement. The data indicates a notable prevalence of representations linking masculinity to the roles of economic provider and protector (61.12%)—i.e., when the man is referred to as a father, father figure, caregiver, savior or economic provider—thus echoing traditional patriarchal notions of masculinity. For instance, in one particular Vodafone advertisement, two daughters are depicted competing for the same object in different scenarios, whether it be a blouse in a clothing store or a remote control in the living room, both exclaiming, “Daaaddd!” The final scene of the advertisement presents the family—comprised of the two daughters, mother, and father—traveling together in their car. Each daughter smiles while using their mobile phones, and the parents sitting in the front seats show a reassuring, calm and happy mood. The ad culminates with the mother turning to the father and saying, leaning her head: “Good one, Dad!” This ending reinforces the family's harmonious dynamic while affirming traditional gender roles subtly and positively.

**Table 3:** Roles and characteristics associated with “being a man” in advertisements with themes aligned with fatherhood, domestic work and care.

**Associates being a man with**	**Number of representations**	***N* (337)**
Economic provider, family man, protector	206	61.12%
Gender roles and traditionally feminine issues	65	19.28%
Artists and celebrities	5	1.48%
A healthy body and physical robustness	3	0.89%
Militarized security and civil protection forces	2	0.59%
Experts, men who study	2	0.59%
Activists, protesters and those engaged in social causes	1	0.29%
	0	0%
Professional role; political, business and/or financial leadership	1	0.29%
Men as victims of harassment and violence (sexual, moral, physical)	0	0%
Sexual desire and sexual performance	0	0%
Toxic, negligent and illicit male behaviors	0	0%
Non-heteronormative	0	0%
Other notions of masculinities	52	15.4%
Total	337	99.93%

Furthermore, this advertisement is emblematic of the way the family is depicted in our corpus: heteronormative, having the man-father in a central position who oversees everything and takes the important decisions—not unsurprisingly, it is the man who drives the car, with the woman on his side and the children behind. As we saw, at the time of the production of these advertisements, Portuguese legislation was redefining the family beyond heteronormativity, and anti-gender ideology sectors were mobilizing against this legislation which they perceived as a threat to the family. By not representing other types of families outside the heteronormative frame, the ads of the corpus condone the resistance to accepting full citizenship for LGBT+.

The second common category focuses on gender roles and traditionally feminine issues (i.e., men presented or associated with activities and gender roles traditionally considered feminine, such as domestic tasks; professions such as hairdressing or related to fashion; etc.), representing 19.28%. Although this percentage is relatively small, its position as second highlights the ongoing potential of advertising for negotiating gender norms in favor of gender equality. One illustrative example of this trend is one of the advertisements for the Continente Baby Fair, which depicts men as engaged in domestic and caregiving responsibilities and tasks using a humouristic tone. From a gender norms negotiation point of view, humor is the key piece in this advertisement as it makes its text and subtext both appealing and relatable without being preachy, thus engaging people who are already sensitive toward the topic of gender equality and contribute to those who are not to be more open to the conversation. A hybrid dimension characterizes this humor, as it derives from the man working so hard in his caregiving role that he becomes tired or forgets something while shopping for the baby. These examples effectively illustrate how this kind of hybrid representation can promote gender (in)equality by highlighting the shared responsibilities of both men and women. Hence, hybridity can address both sides regarding gender (in)equality. As the advertisement emblematically shows, hybridity offers possibilities for hegemonic masculinity to be maintained—by widening its spaces and scope of activities—and even solidified through rhetorical strategies such as humor: the tired father performing “women's tasks” can be read by audiences as a comic figure through a subtext that insinuates that, deep down, men are less gifted for this type of activity historically performed by women. The representation of men associated with notions of gender equality can thus reinforce traditional notions of masculinity.

## 5 Conclusion

This article employed quantitative content analysis to examine how caring masculinities are represented in Portuguese TV advertisements broadcasted in 2015 and how these depictions contribute to deconstruct hegemonic and toxic masculinities and fostering gender equality.

The findings suggest that, although framed within a caring narrative and engaging with themes typically linked to counter-hegemonic masculinity—such as fatherhood and caregiving— analyzed advertisements largely reinforced rather than subverted traditional norms. This occurred as they were embedded within heteronormative family dynamics, which uphold conventional expectations of masculinity, or as they depicted caring practices through aggressive imaginaries, leveraging them as a marketing strategy. The study also identified hybrid representations of masculinity that blend, within a framework of care, traditional and caring masculinities. While this hybridity still sustains patriarchal structures, it also serves as a potential entry point for social change since by incorporating caring masculinities into mainstream media narratives, these hybrid representations introduce alternative ways of being a man, potentially reshaping societal expectations over time.

The analysis of these data suggests that Portuguese TV advertisements reflect a broader societal transition—one in which rigid gender roles are increasingly contested, yet no clear consensus has emerged regarding new masculinities. These findings align with previous research indicating that Portuguese advertising has, since the mid-2010s, both reinforced hegemonic masculinity and created space for evolving representations of masculinity, namely caring masculinities (e.g., Januário, 2016; Santos and de Carvalho, 2018).

By providing main trends in the representation of caring masculinities in TV ads in 2015 in Portugal, this study provides a foundation for future comparative studies that examine how masculinity and femininity have been negotiated in Portuguese advertisements over the past decade, exploring whether caring masculinities are depicted in less hybridized forms. A comparative analysis would provide deeper insights into media's evolving role in shaping gender norms, furthering our understanding of negotiations and transformation of masculinities in contemporary western semi-peripheral societies.

## Data Availability

The original contributions presented in the study are included in the article/supplementary material, further inquiries can be directed to the corresponding author.

## Footnotes

1 The first points to “the configuration of gender practice (…) which guarantees (or is taken to guarantee) the dominant position of men and the subordination of women” (Connell, 2005, p. 77)”—and, in fact, is depicted as superior, dominant and the absolute opposite of femininity—the second refers to men who are “easily assimilated to femininity” (e.g. homosexual men), and the third highlights characteristics such as “race” for example and the fact that inclusion or exclusion from the ideal type of masculinity “is always relative to the authorization of the hegemonic masculinity of the dominant group” (2005, p. 80, 81). Marginalized masculinities refer, for example, to black masculinities in white supremacy contexts.

2 E.g., body creams, shampoos, skin care products, deodorants.

3 E.g., alarms, insurance, and products or services for maintaining or repairing homes, gardens, cars, or motorcycles.
